# Extreme Salinity Change Governs Microbial Community Assembly and Interactions

**DOI:** 10.1111/1758-2229.70301

**Published:** 2026-02-15

**Authors:** Christopher Keneally, Virginie Gaget, Daniel Chilton, Tyler N. Dornan, James Hensel, Ashleigh E. Keneally, Stephen P. Kidd, Justin D. Brookes

**Affiliations:** ^1^ School of Biological Sciences, College of Science Adelaide University Adelaide South Australia Australia; ^2^ Department of Surgery, Adelaide University, the Queen Elizabeth Hospital Adelaide South Australia Australia; ^3^ Basil Hetzel Institute for Translational Health Research, Central Adelaide Local Health Network Woodville South Australia Australia; ^4^ College of Science and Engineering Flinders University Adelaide South Australia Australia; ^5^ Research Centre for Infectious Disease (RCID), Adelaide University Adelaide South Australia Australia; ^6^ Australian Centre for Antimicrobial Resistance Ecology (ACARE), Adelaide University Adelaide South Australia Australia

**Keywords:** coastal lagoon, community assembly, hypersaline, microbial ecology, network analysis

## Abstract

Coastal wetlands are highly vulnerable to climate‐driven salinisation, which reshapes critical microbial processes underpinning nutrient cycling and energy flow. We examined how sediment microbial communities vary with salinity across the Coorong Lagoon (South Australia), spanning estuarine (0–40 g L^−1^), intermediate (40–100 g L^−1^) and hypersaline (100–150 g L^−1^) waters. Salinity was found to be the dominant driver of sediment microbial community composition, diversity and assembly. High salinity favoured specialists and homogenous community structures, with generalist bacteria persisting across intermediate salinities and supporting ecosystem resilience. Sulfur and carbon cycling is likely dependent on salinity, as bacterial sulfur‐oxidisers were abundant estuarine specialists, whereas methane producers (Archaeal methanogens) and sulfate‐reducers were enriched at high salinity. Deterministic microbial community assembly (homogeneous selection) was dominant, increasing at extreme salinity, which acted as a strong environmental filter. Community complexity increased at both high and low salinity ranges, with intermediate salinity exhibiting less complexity, suggesting community reorganisation under osmotic stress. The varied roles of specialists and generalists at different salinities support ecosystem function, where increased heterogeneity and specialisation in hypersaline conditions suggest vulnerability of the community to disturbance. These findings provide insight into how microbially underpinned ecosystems may respond to future climate‐driven salinisation, important for making predictions and informing mitigation strategies.

## Graphical abstract

Along the Coorong Lagoon's extreme salinity gradient, hypersalinity deterministically enriches halophilic specialists and reorganises sediment microbial networks. Network complexity peaks at salinity extremes, revealing pivotal ecological trade‐offs that will steer nutrient cycling and lagoon resilience as coastal wetland salinisation accelerates.

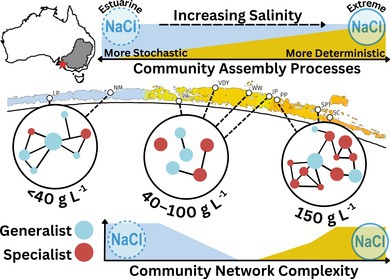

## 1 Introduction

Coastal lagoons are dynamic, shallow systems separated from the sea by sand bars, where salinity variability is governed by the balance between freshwater inflows and tidal exchange (Barnes 1980; Soria et al. 2022). Coastal lagoons are definitionally similar to estuaries, both being “semi‐enclosed inland water bodies with connections to the sea (Kjerfve 1986),” but lagoons can be separated by their increased water residence time and slowed out‐flow (Schubert and Telesh 2017), with variably restricted connection to the sea. Coastal lagoon ecosystems are globally distributed, representing 13% of the world's coastline and providing critical habitats for highly diverse biota and valuable ecosystem services (Barnes 1980; Carrasco et al. 2016; Newton et al. 2018), which are critically underpinned by their microbial ecology (Keneally, Gaget, et al. 2025). However, the stability and health of such coastal ecosystems is threatened by potential climate change impacts, decreasing freshwater flows, and eutrophication (Eisenreich et al. 2005; Keneally, Gaget, et al. 2025; Tweedley et al. 2019). Salinity in many coastal lagoons is increasing due to evapoconcentration of salts, accompanied by rising temperatures and changes in regional precipitation patterns (Tweedley et al. 2019). As a result, significant selection pressures may be imposed on resident organisms, necessitating halotolerance adaptations, with uncertain consequences for overall ecosystem‐scale processes (Brookes et al. 2009; Chilton et al. 2021).

Understanding the dynamics of resident microbial communities is essential for understanding ecosystem processes in hypersaline coastal lagoons (Keneally, Gaget, et al. 2025; Reed et al. 2014). Salinity acts as an important environmental filter across many different ecosystem types, limiting the survivability of some microorganisms while favouring others that have adapted to salinity stress (Oren 2024; Rath et al. 2019; Welsh 2000). Organismal salinity preference is a deeply conserved trait (Martiny et al. 2015), requiring significant phylogenetic adaptation of microbial communities and leading to trade‐offs in community function with implications for broader ecosystem processes (Baker et al. 2024; Dupont et al. 2014; Shu and Huang 2022). For example, salinity significantly affects decomposition and nutrient cycling in soils, inland waterways and coastal aquatic systems (Chowdhury et al. 2011; De León‐Lorenzana et al. 2018; Keneally, Gaget, et al. 2025; Wang et al. 2018; Yang et al. 2016; Zhang et al. 2021).

The mechanisms of microbial biogeography can be explained by community ecology frameworks like niche theory (Austin 1985), and neutral theory (Hubbell 2011), which offer valuable insights into how environmental pressures shape ecosystems (Vellend 2010). These frameworks help delineate the roles and relative importance of deterministic processes (i.e., niche‐based selection where specific environmental and biotic factors favour certain taxa) and stochastic processes (i.e., random dispersal and drift) in community assembly (Kraft et al. 2015). In hypersaline coastal lagoons, deterministic forces are expected to stem primarily from steep, persistent salinity and nutrient gradients, whereas stochasticity is likely driven by episodic freshwater inflows, or wind‐driven resuspension (Chilton 2024).

Deterministic processes of selection are considered as either homogenous (consistent environmental conditions and reduced community turnover, favouring habitat specialists) or heterogeneous (fluctuation in environmental conditions and increased community turnover, favouring habitat generalists). Additionally, stochastic processes can be classified as having homogenous dispersal (high dispersal rate and reduced community turnover) or being limited to dispersal (low dispersal rate and increased community turnover) (Stegen et al. 2012). Salinity increases deterministic selection of microbes in coastal wetland soils and sediments, particularly enriching halophilic Bacteria and Archaea (Yu et al. 2022; Zhang et al. 2021). However, stochastic processes can also dominate sediment microbial community assembly in hypersaline salterns (Song et al. 2022).

Coastal lagoons with dynamic salinity gradients present unique opportunities to study microbial niche‐adaptation and community assembly in response to fluctuating salinity levels. Conditions within these ecosystems provide interesting insight into niche‐adaptation from microorganisms, wherein ‘specialist’ and ‘generalist’ taxa are selected based on their ability to inhabit a narrow or broad range of habitat, respectively (Chen et al. 2021; Hutchinson 1978; Pandit et al. 2009). This niche specialisation approach shows increased robustness to detecting ‘winners and losers’ in ecological disturbance analyses, relative to traditional richness and diversity analyses (Devictor and Robert 2009). Generally, habitat generalist community dynamics are governed by more stochastic dispersal processes, while habitat specialists are subject to deterministic selection processes (Pandit et al. 2009). In highly dynamic salinity zones, regular disturbances may prevent habitat specialisation, potentially favouring halotolerant generalists (Devictor and Robert 2009). In contrast, stable conditions at salinity extremes may select for halophilic or estuarine habitat specialists. Understanding these patterns can shed light on the mechanisms of species coexistence and the balance between specialists and generalists in response to environmental stressors.

This study investigates how salinity influences prokaryotic microbial community composition, diversity, assembly processes, and inter‐taxa relationships within the Coorong. The Coorong is a coastal lagoon in South Australia characterised by a wide salinity gradient from freshwater to hypersaline conditions (Brookes et al. 2023), which fluctuates depending on freshwater flows (Brookes et al. 2022). Of particular interest in the Coorong region are microbial interactions that alter biogeochemical processes such as denitrification (Cook et al. 2009; Mosley et al. 2023; Huang et al. 2025) and methane cycling (Keneally, Chilton, et al. 2025; Keneally, Southgate, et al. 2024), as these affect water quality and greenhouse‐gas emissions at both local and global scales. Here, it is hypothesised that salinisation drives niche‐based selection, decreasing overall community diversity and increasing the competitive advantage of halophilic habitat specialists over halotolerant generalists. Further, it is hypothesised that regular disturbances in dynamic salinity zones favour habitat generalists, whereas stable communities dominated by specialists are favoured at salinity extremes.

Understanding these changes is critical, because alterations in the balance of halophilic and halotolerant microorganisms can affect key biogeochemical processes (i.e., nutrient and carbon cycling) potentially influencing carbon storage/emissions, and overall ecosystem stability, especially as salinity stress intensifies under climate change and anthropogenic pressures (Oren 2008; Tweedley et al. 2019).

## 2 Methods

### 2.1 Site Description

**FIGURE 1:**
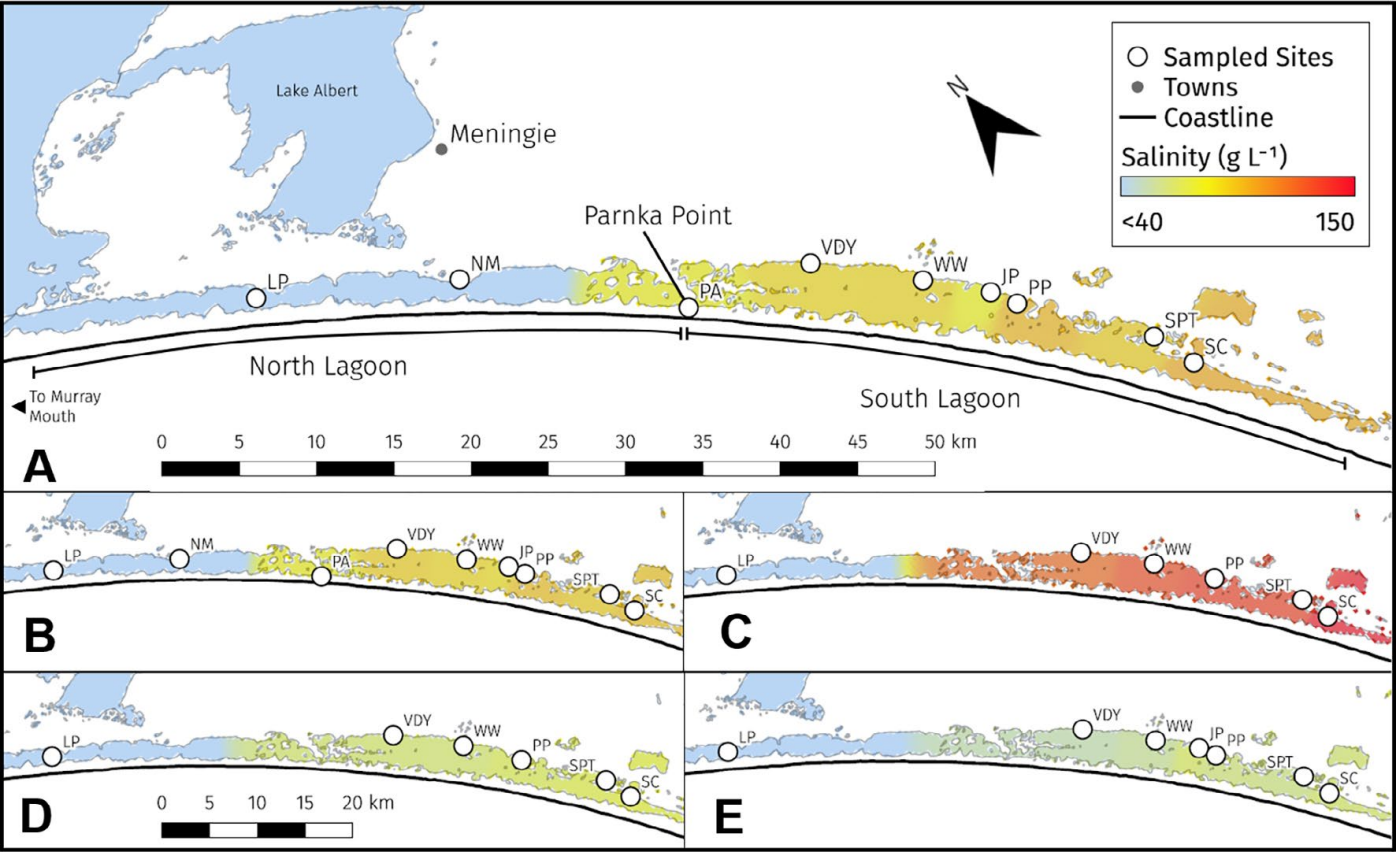
Map of study sites. Salinity is represented in colour gradients for (A) mean values across all samples, (B) Summer sampling trips, (C) Autumn sampling trips, (D) Winter sampling trips and (E) Spring sampling trips. Blue colouring represents salinity < 40 g L^−1^. Red colouring represents maximum recorded salinity levels. JP, jack point; LP, long point; NM, Noonamena; PA, Parnka point; PP, policeman point; SC, salt creek; SPT, snipe point; VDY, villa Dei Yumpa; WW, woods well.

The Coorong is characterised by three distinct eco‐regions: the Murray Mouth, North Lagoon, and South Lagoon (Figure 1). The Coorong has maximum depths of 3–4 m in winter (Noye 1974), with an average water depth of 1 m (Geddes and Butler 1984). The two lagoons are connected by a narrow channel near Parnka Point, where flow between lagoons is constricted and intensifies the salinity gradient which increases south‐eastward from Lake Alexandrina to Salt Creek (Figure 1, Table S1). The Coorong is separated from the freshwater Lake Alexandrina by five barrages that maintain upstream freshwater conditions in the lake and force unidirectional freshwater flow to the Coorong and Southern Ocean (Mosley et al. 2018). The North Lagoon is tidally influenced and mudflats are inundated and exposed daily depending on tide and wind. The Coorong, Lower Lakes (Lake Alexandrina and Lake Albert), and Murray Mouth (CLLMM) region is recognised as a Wetland of International Importance under the Ramsar Convention as an important habitat for waterbirds (Jackson et al. 2024), supporting more than 1% of the global populations of nine waterbird taxa (Paton et al. 2009). It serves as an important waterbird refuge, hosting up to 90% of waterbirds counted at The Living Murray ‘icon’ sites in the Murray‐Darling Basin during drought (Kingsford and Porter 2008).

### 2.2 Environmental Variables

Triplicate measurements of in situ physico‐chemical characteristics were collected at 50–100 mm above sediments at each sampled site using a YSI EXO2 multiparameter sonde (Yellow Springs Instruments, USA). These included salinity, water temperature, dissolved oxygen concentration (DO) and pH.

### 2.3 Sediment Coring and Organic Matter Calculations

Coorong surface sediments were sampled along a salinity gradient, intermittently, over a 4‐year period from 2019 to 2022 (Figure 1). Sampling was conducted across southern hemisphere seasons, defined here as: summer (1 December–28/29 February), autumn (1 March–31 May), winter (1 June–31 August) and spring (1 September–30 November). Triplicate sediment cores were collected at each site in PVC tubes (internal diameter 57 mm). The upper 0–40 mm of each core was isolated and homogenised with a sterile metal spatula, of which, 3 mL was stored in DNAse/RNAse‐free tubes (Greiner). To prevent nucleic acid degradation, sediments were preserved in 9 mL of *LifeGuard* preservation solution (Qiagen) and stored at −20°C in a portable freezer. To account for sample contamination during processing, field negative controls were also taken in situ, by filling a sterile 15 mL tube with 3 mL *LifeGuard* solution, then rinsing the disinfected spatula used for core collection with 1 mL nuclease free water (Qiagen). *LifeGuard* preservation solution was chosen as it is effective in maximising environmental nucleic acid yields and quality in sediments along broad salinity gradients of the Coorong (Keneally, Gaget, et al. 2024).

Separate sediment cores were collected to calculate sediment organic matter (OM) content, from which sediment aliquots (~12 g) were taken from the upper 0–40 mm portion. Aliquots were dried to a constant weight at 60°C, and the OM was identified as the difference in dry weight after combustion to a constant weight at 400°C. In both cases, constant weight was determined by regularly weighing samples until daily weight loss plateaued at < 4%.

### 2.4 DNA Extraction, Amplification and Sequencing Methods

DNA was extracted from sediments using the PowerSoil Kit (Qiagen), following kit instructions. To account for potential contamination introduced during laboratory processing, additional ‘no sample’ controls were included with each extraction batch. Extract concentration was measured with a Qubit (Thermo Fisher Scientific). All controls were below the Qubit quantitation limit but were retained for later identification of contaminant sequences.

Briefly, 16S rRNA genes (from the V3‐V4 hypervariable region) were amplified from sediment DNA using the universal prokaryote primer set: 341F (forward) 5′‐CCTAYGGGRBGCASCAG‐3′ and 806R (reverse) 5′‐GGACTACNNGGGTATCTAAT‐3′ at the Australian Genome Research Facility (AGRF). Thermal profiles for first stage PCR were: 95°C and 29 cycles of 30″–94°C, 60″–50°C, 60″–72°C, with a final extension of 7′–72°C. Secondary PCR was performed to index amplicons and attach sequencing adapters as described in the Nextera XT Index kit (Illumina). The resulting amplicons were sequenced following the procedure detailed in Keneally, Southgate, et al. (2024). The equimolar pool of amplicons was then sequenced on the MiSeq platform (Illumina), using 2 × 300 bp paired‐end read chemistry.

Samples were demultiplexed by AGRF using Illumina scripts. Raw fastq files had primers trimmed and were filtered for quality, along with combining forward and reverse reads using DADA2 (Callahan et al. 2016) in QIIME 2 (version 2023.9) (Bolyen et al. 2019). Taxonomy was then assigned to the Amplicon Sequence Variants (ASVs) with a classifier trained on Silva v138 SSU 99% reference sequences.

Further analysis of the data was conducted with R statistical software (version 4.4.0) (R Core Team 2021). Twenty‐eight contaminant ASVs were identified and removed with *decontam* (Davis et al. 2018) (*method: prevalence, threshold: 0.5*) (45,384 → 45,356 ASVs). Chloroplast and mitochondrial reads were also removed with *phyloseq* (McMurdie and Holmes 2013) (*subset_taxa*) (44,747 ASVs).

### 2.5 Statistical Methods

#### 2.5.1 Specialist/Generalist Classification

To classify taxa as specialists or generalists, taxa were first agglomerated to genus rank with the *microbiome* R package (Lahti and Shetty 2017). Random sub‐sampling was then performed below the minimum library size (11,400 reads), without replacement, resulting in a total of 1343 Genera. To ensure robust classification, Shannon diversity, Occurrence, and Levin's niche width indexes were calculated over 1000 random permutations and each taxon's deviation from null values compared. Levin's niche width *B*_*n*_[*j*] is calculated as follows (Equation 1):

(1) $$B_{n}\left[j\right]=\frac{1}{R\sum p{\left[i\right]}^{2}}$$

where *R* is the number of environments, and *p[i]* is the proportion of taxon *j* in environment *i*. Then, Genera were classified into either: (1) Generalists (where *B*_*n*_ > 95% CI), and (2) specialists (where *B*_*n*_ < 95% CI), or (3) non‐significant (where *B*_*n*_ was within 95% CI). Where un‐agglomerated ASV tables were used, generalist and specialist classifications were extrapolated from agglomerated data and assigned to matching Genera.

#### 2.5.2 Community Diversity and Identification of Important Taxa

Salinity's relative importance among environmental variables was assessed using PERMANOVA (*vegan::adonis2*) on Bray‐Curtis dissimilarity distances of the microbial community (Dixon 2003). Salinity and other measured environmental variables (temperature, pH, dissolved oxygen, sediment organic content) were first modelled as continuous covariates. Categorical salinity classes and seasonality (and their interaction) were tested separately in a factorial PERMANOVA to avoid conflating continuous‐covariate and factor‐based models. To confirm inference was not driven by rare taxa, the PERMANOVA was repeated after filtering low‐abundance (total relative abundance ≥ 1 × 10^−4^) and low‐prevalence taxa (present in ≥ 3 samples); results were unchanged (Δ*R* ^2^ < 0.001; 999 permutations). Multicollinearity among numeric predictors was negligible (VIF: 1.06–1.22).

To further distinguish salinity‐driven from biogeographical effects on microbial diversity, geographic distances between samples were calculated and paired with Bray‐Curtis β‐diversity similarities. Distance‐decay relationships were plotted against geographic distance and salinity variation, partitioned by season to account for temporal bias. Scaled salinity data were used to compute Euclidean distances for assessing their effect on community similarity, and linear regressions evaluated the influences of biogeography and salinity across seasons.

To test seasonality and categorical salinity‐group effects on microbial communities, 3 salinity groupings were defined, based on trophic criteria categories (Por 1980) (0–40 g L^−1^, 40–100 g L^−1^ and 100–150 g L^−1^). PERMANOVA tests were performed with salinity group, season, and their interaction (salinity‐group × season) for the whole microbial community, generalists and specialists. Alpha diversity metrics were calculated from unfiltered sequence data and differences between salinity categories were identified with Wilcoxon tests.

Random forest modelling was conducted in parallel across 500 bootstrap iterations to determine the importance of each taxon in classifying the three salinity groups. Mean decrease in Gini index (how well a variable splits the data into groups) was calculated for each taxon to assess importance, and the top 20 generalists and specialists were selected. For each taxon, bootstrap mean importance and 95% confidence intervals were estimated from the bootstrap distribution. Model robustness was assessed using out‐of‐bag (OOB) prediction from an independent forest. Relative abundance of important taxa was calculated on a per‐sample basis as each taxon's read count divided by the total reads.

#### 2.5.3 Community Assembly Process Determination

To determine whether microbial communities in the study site are structured by deterministic processes (specifically, homogeneous or heterogeneous selection by biotic and abiotic factors) or stochastic processes (random cell turnover or dispersal events), a whole‐community null‐model approach was taken (Stegen et al. 2013, 2012). Briefly, the β nearest‐taxa index (βNTI) and Bray‐Curtis‐based Raup‐Crick (RC_bray_) metrics were calculated for pairwise community comparisons from un‐agglomerated ASV tables. The βNTI measures deviations in β mean‐nearest‐taxon‐distance (βMNTD) from a null‐model (999 randomisations), identifying deterministic processes significantly deviating from null (heterogeneous selection: |βNTI| > 2, or homogeneous selection: |βNTI| < −2). The RC_bray_ value further categorises stochastic assemblies falling between |βNTI| −2 and 2 (Zhou and Ning 2017). These include dispersal limitation (RC_bray_ > 0.95), homogenising dispersal (RC_bray_ < −0.95) and undominated processes not significantly deviating from the null (RC_bray_ between −0.95 and 0.95) (see Figure S1 for conceptual diagram).

#### 2.5.4 Network Analysis and Community Interactions

Microbial correlation networks were constructed from un‐agglomerated ASV tables with *SpiecEasi* (Kurtz et al. 2015), and their network‐level topology statistics were compared between subnetworks constructed for each salinity category. To increase robustness and reduce the effect of spurious correlations arising from rare species, taxa below 0.01% abundance were removed from the data prior to network construction (Berry and Widder 2014). Networks were visualised, and node‐level network topology statistics were calculated with *Gephi* (version 0.10) (Bastian et al. 2009).

## 3 Results

### 3.1 Environmental Variables

Salinity in the Coorong exhibits a clear spatial gradient, persisting across seasons (Figure 1, Table S1). The highest values were observed in South Lagoon sites (Figure 1: VDY, WW, PP, SPT and SC) during autumn. Salinity declined rapidly alongside decreasing temperature through winter and spring (Figure 1, Table S1). The overall salinity gradient remained a defining feature of the system throughout the sampling period (Figure 1, Table S1). Despite increased autumn salinity, average bottom water temperatures were slightly higher in summer. Bottom water DO was highest in North Lagoon sites (LP, NM), whereas DO concentrations were very low (< 5 mg O_2_ L^−1^) in some South Lagoon sites (VDY, WW, SPT) in autumn and winter. Salinity reduces the solubility of oxygen in water, leading to the lowest average pH and DO levels (3.95–4.92 mg O_2_ L^−1^) observed in all South Lagoon sites during autumn (Table S1).

### 3.2 Specialist/Generalist Classification

Out of 1343 identified genera, 458 taxa (34.1%) were classified as specialists and 132 taxa (9.8%) as generalists using a triple classification method (Figure S2). Therefore, across all salinity ranges in the Coorong, specialists were found to be more numerous than generalists.

### 3.3 Community Diversity

PERMANOVA analyses on continuous environmental variables revealed salinity as the most influential driver of microbial community structure (PERMANOVA *R* ^2^: 0.21, *p* < 0.01). Both water temperature and pH also had significant effects, with much less variance explained (*R* ^2^: 0.06 and 0.05, respectively, *p* < 0.01). Sediment organic content contributed an even smaller portion of variance (*R* ^2^: 0.03, *p* < 0.05), and DO had no significant effect on sedimentary microbial community structures (Table 1).

**TABLE 1:** Permutational multivariate analysis of variance (PERMANOVA; *vegan::Adonis2* distance based linear model on continuous covariates) results examining the effects of continuous environmental variables on microbial community composition, based on Bray‐Curtis distances.

Environmental variable	Df	SS	*R* ^2^	*F*	*p*
Salinity (g L^−1^)	1	2.3673	0.2107	20.9211	**< 0.001**
Sediment organic content (%)	1	0.2998	0.0267	2.6494	**0.014**
Dissolved oxygen (mg O_2_ L^−1^)	1	0.1747	0.0156	1.5442	0.162
Water temperature (°C)	1	0.6487	0.0577	5.7330	**< 0.001**
pH	1	0.6160	0.0548	5.4435	**< 0.001**
Residual	63	7.1288	0.6345		
Total	68	11.2353	1.0		

*Note:* Significant *p* values (*p* < 0.05) are bolded.

Abbreviations: Df, degrees of freedom; *F*, pseudo‐*F* statistic; *R* ^2^, proportion of variance explained; SS, sum of squares.

**FIGURE 2:**
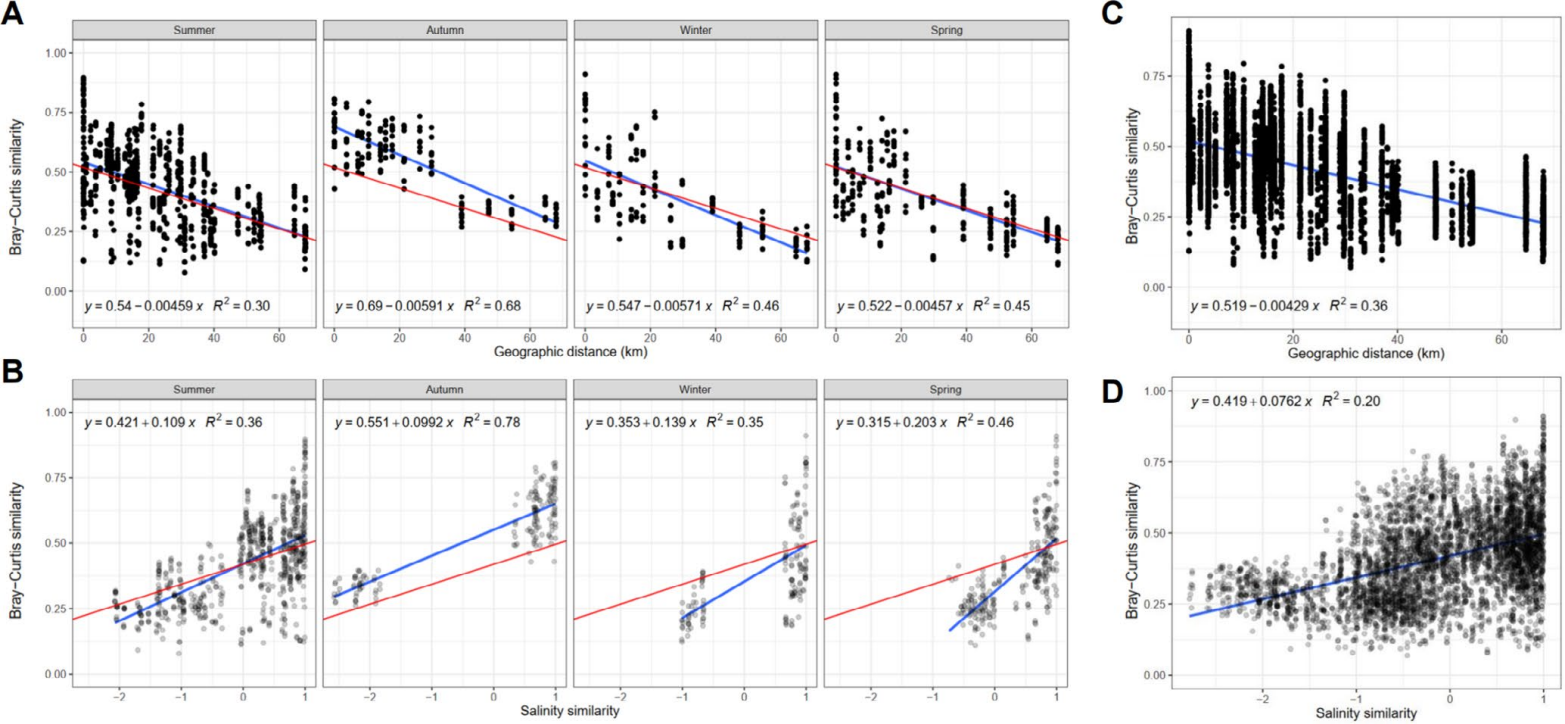
Regressions of between‐sample Bray‐Curtis whole‐community similarity with spatial distribution and salinity similarity. (A) Spatial distribution and (B) salinity similarity faceted by season. (C) Spatial distribution and (D) salinity similarity with all seasons combined. Blue regression lines show linear fit for subset, while red lines indicate the full regressions shown in (C) and (D). All linear models pass hypothesis testing with *p* values not exceeding 5.67 × 10^−6^.

Positive correlations between community and salinity were found in all cases (Figure 2B,D whole‐community slope: 0.0762). Sites with similar salinity shared similar communities, particularly among habitat specialists (Figures S3D and S4D, generalists slope: 0.0242, specialists slope: 0.136). This indicates that salinity is a key driver of community composition, especially for specialists, adapted to specific salinity conditions.

**FIGURE 3:**
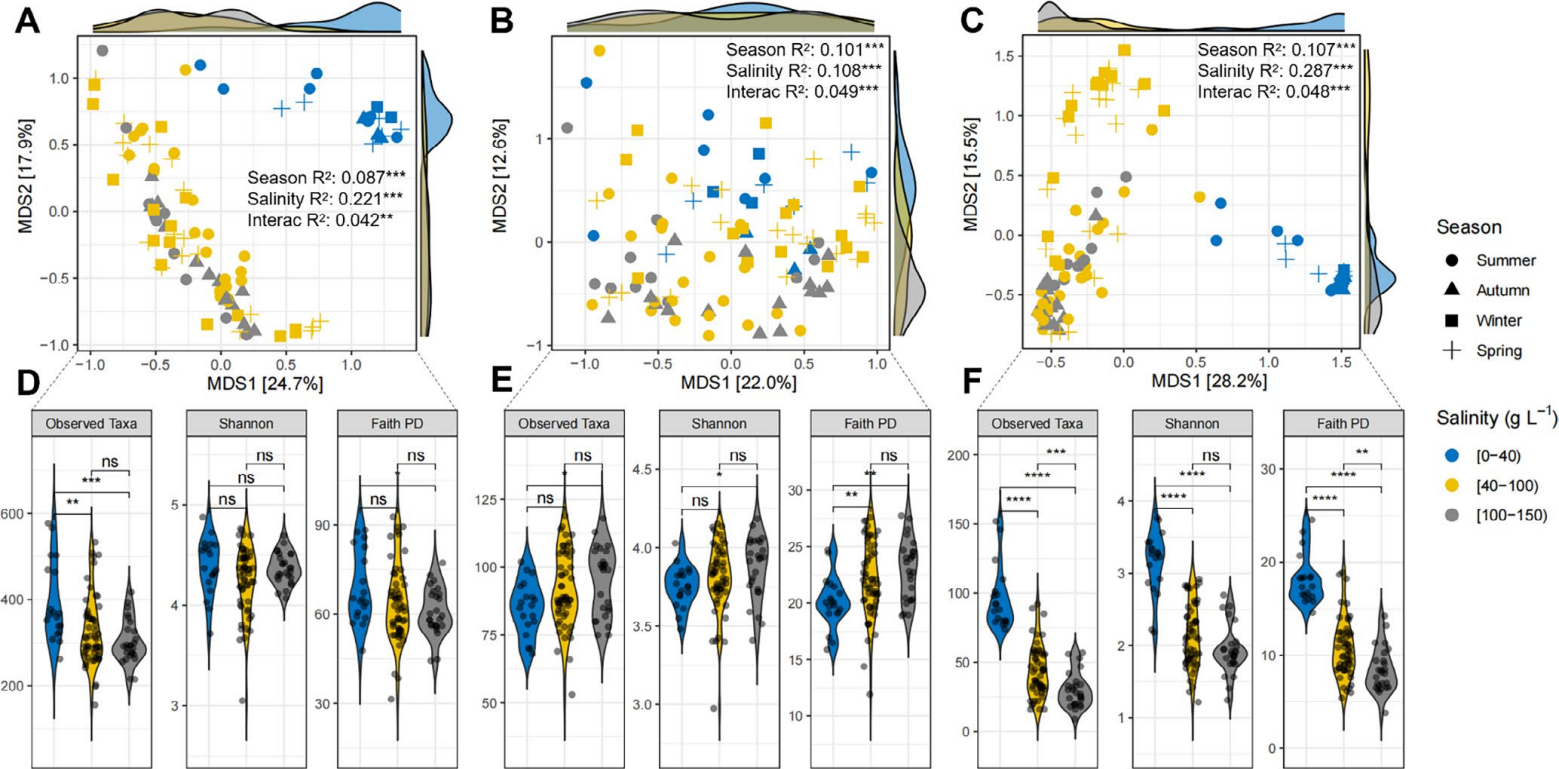
Microbial diversity. Multidimensional scaling (MDS) plots show β‐diversity based on Bray‐Curtis dissimilarity of (A) whole community, (B) generalists and (C) specialists. Significant effects of salinity category, season, or the interaction of salinity × season categories were detected by PERMANOVA. Violin plots show α‐diversity differences across the same 3 salinity categories: (D) whole community, (E) generalists and (F) specialists. Significant differences in richness (Observed Taxa), Shannon diversity and phylogenetic diversity (Faith PD) were detected by Wilcoxon tests. *: *p* < 0.05, **: *p* < 0.01, ***: *p* < 0.001, ****: *p* < 0.0001.

Specialists clustered more distinctly into salinity groups than the whole‐community, showing stronger relationships with salinity (Figure 3C vs. 3A), suggesting that specialists are more sensitive to salinity changes and lead to greater differentiation in community composition across the salinity gradient (PERMANOVA *R* ^2^: 0.29 for specialists and 0.11 for generalists). Seasonality affected groups relatively equally (9%–11%). Although the interaction between seasonality and salinity was significant, it accounted for only 4%–5% of variation in each case (Figure 3A–C).

Overall, taxa richness and phylogenetic diversity decreased significantly with increasing salinity (Figure 3D). Specialists experienced significant reductions in all alpha diversity metrics above 40 g L^−1^ (Figure 3F), indicating that high salinity conditions limit the diversity of specialists. In contrast, generalists showed increases in richness and Shannon diversity between salinity extremes (Figure 3E), suggesting that they can thrive across a broader range of conditions.

### 3.4 Identification of Important Taxa

**FIGURE 4:**
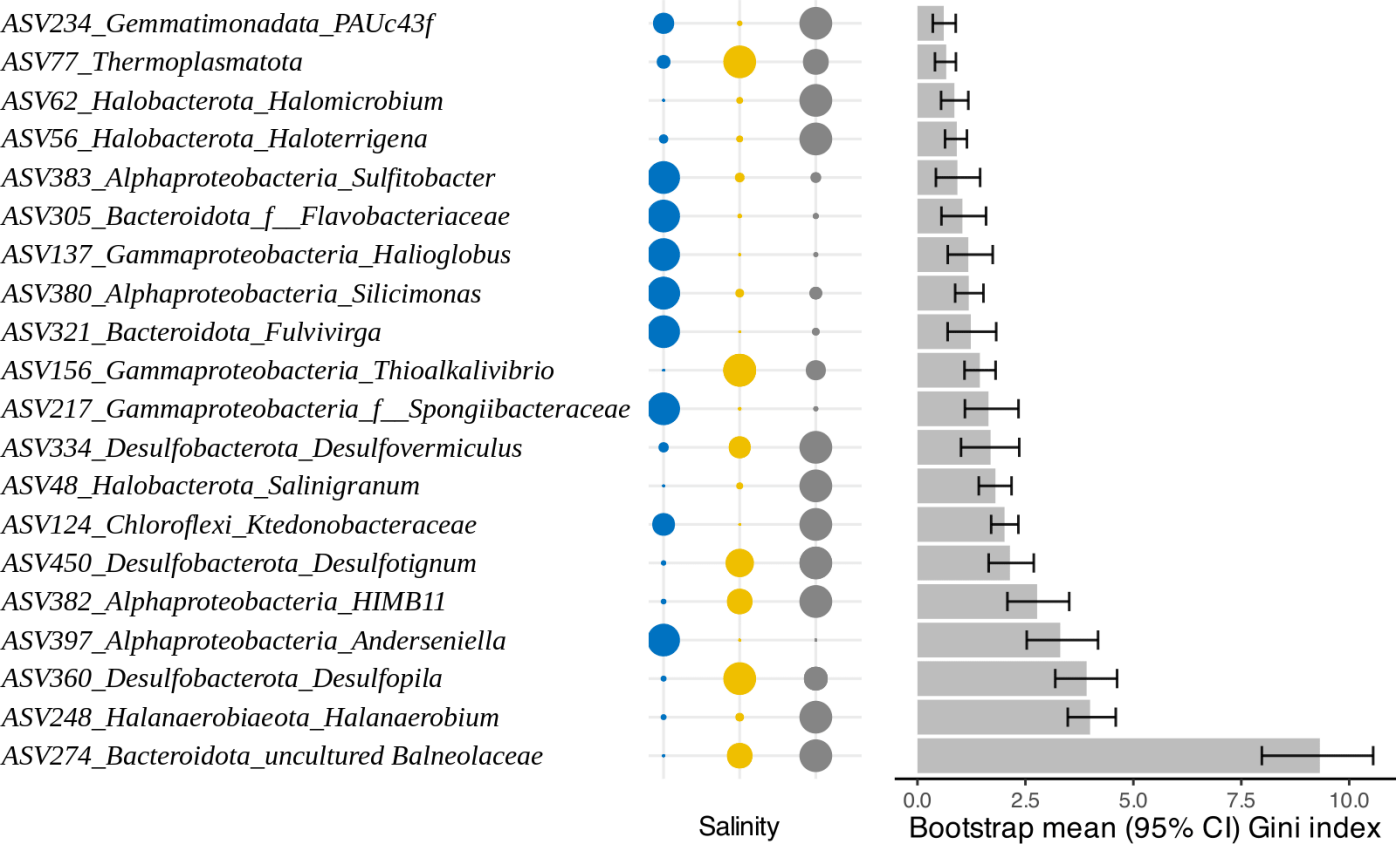
Top 20 specialist taxa discriminating between salinity categories. For each taxon, taxonomy is displayed at both Phylum level and the deepest classified level. Dot plots indicate the relative abundance by salinity category (dot size is proportional to relative abundance per‐sample), distributed along the *x* axis and represented by colour, as follows: Blue: 0–40 g L^−1^, yellow: 40–100 g L^−1^, grey: 100–150 g L^−1^. The horizontal bar graph shows the mean importance of each taxon as predicted by bootstrapped random forest modelling (bootstrap mean with 95% confidence intervals).

Random forest modelling identified the ‘most important’ taxa discriminating between salinity categories. Model performance was stable (mean OOB accuracy = 0.867 ± 0.020 SD across bootstrap runs; final‐model OOB accuracy = 0.879; balanced accuracy = 0.886). In hypersaline conditions, important specialists included *Balneolaceae* (Bacteroidetes), *Halanaerobium* spp. (Halanaerobiaeota) and *Desulfopila* spp. (Desulfobacterota). Significant Archaeal specialists included halophilic Halobacterota (i.e., *Salinigranum* spp., *Haloterrigena* spp., *Halomicrobium* spp.) enriched at 100–150 g L^−1^, and Thermoplasmatota at 40–100 g L^−1^ and above (Figure 4). Of the specialists important for discriminating salinity niches, not all were halotolerant; some Bacteroidetes and Proteobacteria were scarce above 40 g L^−1^. These marine/estuarine specialists included *Anderseniella* spp., *Spongiibacteraceae*, *Silicimonas* spp., *Halioglobus* spp., *Fulvivirga* spp., *Flavobacteraceae* and *Sulfitobacter*. Notably, *Ktedonobacteraceae* (Chloroflexi) and Gemmatimonadota group PAUC43f were most abundant at salinity extremes but absent at intermediate levels (Figure 4). The presence of these taxa in lower salinity conditions highlights the diversity of specialist adaptations along the salinity gradient.

Generalists showed minimal abundance differences across salinity groups and were dominated by Proteobacteria, Actinobacteria and Bacteroidetes (Figure S5). The top three generalists were Marinimicrobia (SAR406), *Oceanicaulis* and *Oleiphilus*.

### 3.5 Community Assembly Process Determination

**FIGURE 5:**
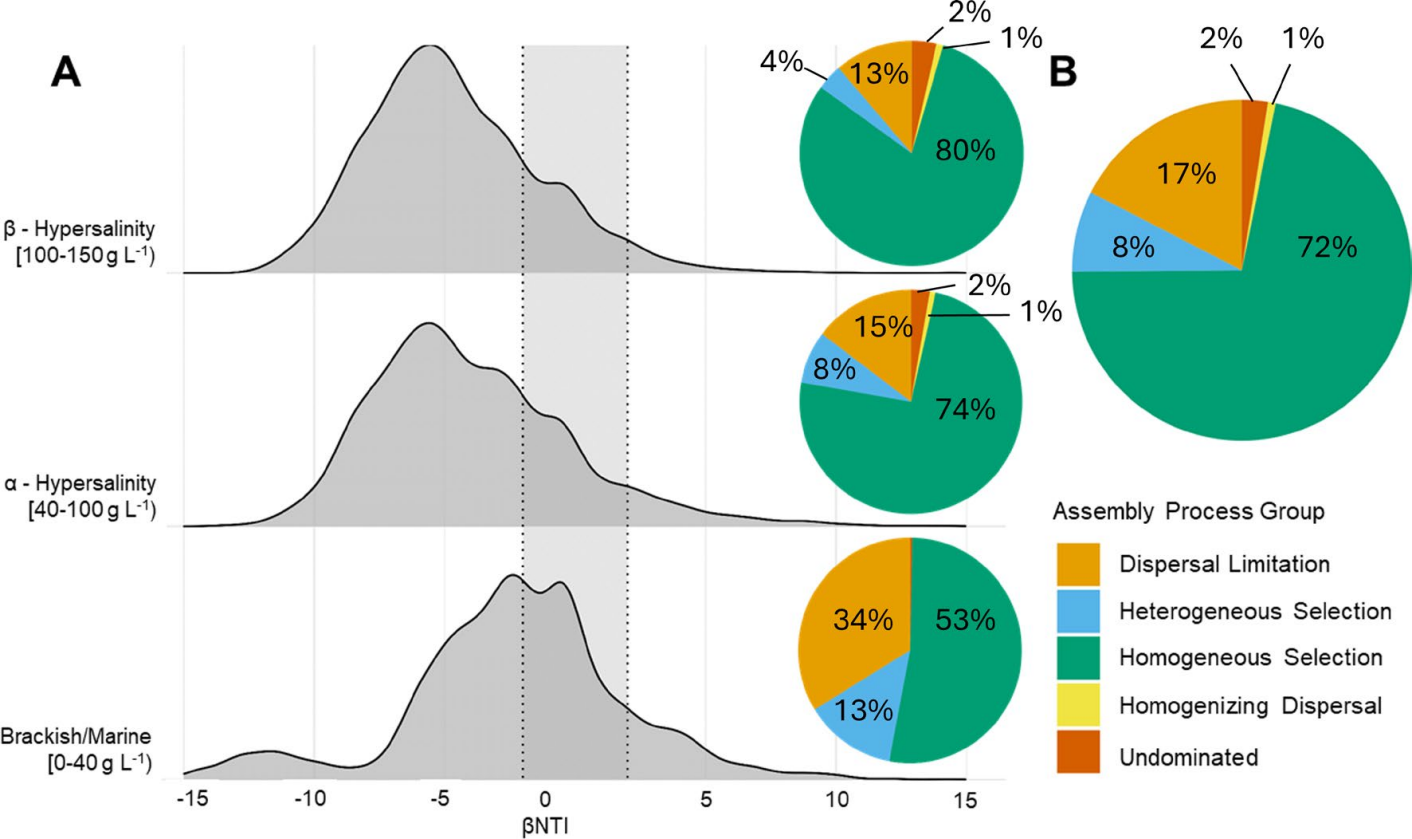
Relative importance of community assembly processes structuring sediment microbial communities. (A) Variation in β nearest‐taxa index (βNTI) between salinity categories. (B) Overall distribution of community assembly processes. Ridge plots display the distribution of βNTI values across each salinity category. Shaded areas flanked by dotted lines indicate significant deviation of βNTI from null, while pie charts represent the relative contribution of stochastic (dispersal limitation, homogenising dispersal and undominated), and deterministic (heterogeneous/homogeneous selection) assembly processes.

Overall, community assembly was mainly dominated by homogeneous selection by deterministic processes (72%), and dispersal limitation by stochastic processes (17%) (Figure 5B). While deterministic processes were dominant across salinity categories, an increasing magnitude of selection processes with increasing salinity was observed, with homogenous selection steadily increasing from low salinity (53%) to intermediate (74%), and high salinity (80%, Figures 5A and S7).

Stochastic processes, particularly dispersal limitation, were highest at low salinity (34%), and progressively decreased with increasing salinity (Figure 5A). Heterogeneous selection processes also decreased along the salinity gradient (13%, 8% and 4% in low, intermediate and high salinity, respectively Figure 5A). At lower salinities, random dispersal and chance events play a larger role in shaping communities, in comparison to higher salinities, where environmental filtering by salinity (homogenous selection) becomes the overwhelmingly dominant force. This shift reflects the increasing importance of niche‐based selection in more extreme environments.

Similar patterns were also observed in site‐based analyses of community assembly, along the salinity gradient (Figure S6). In almost all sites, homogeneous selection of deterministic assembly was the dominant community assembly process, with increasing importance along the salinity gradient. An exception was observed at Noonamena (NM, Figure 1) where the stochastic process of dispersal limitation dominated community assembly (60%, Figure S6A).

### 3.6 Network Analysis and Community Interactions

Network analyses suggested that with increasing salinity, microbial communities became more interconnected and complex (Table 2; Figure S8). The number of nodes and edges increased, along with average degree and graph density, indicating denser interactions among taxa. Across the 0–40 g L^−1^, 40–100 g L^−1^ and 100–150 g L^−1^ categories, negative edges rose from 38% to 43%, whereas positive edges fell from 62% to 57%, indicating a shift toward competition under hypersaline stress (Table 2). Higher average degree suggests that taxa are interacting with more partners, and increased graph density indicates that a higher proportion of possible connections are realised in the network (Table 2).

**TABLE 2:** Network‐level topological microbial community network features across salinity categories (g L^−1^).

Network properties	[0–40]	[40–100]	[100–150]
Number of nodes	1323	1384	1349
Number of edges	14,187	15,209	16,767
Average degree	21.447	21.978	24.858
Average path length	2.806	2.725	2.725
Network diameter	6	6	6
Average clustering coefficient	0.115	0.055	0.089
Average eigenvector centrality	0.064	0.035	0.028
Modularity	0.979	0.82	1.067
Graph density	0.016	0.016	0.018
Positive edges			
Total positive	61.57%	62.46%	56.64%
Generalist‐involving	13.35%	20.43%	21.59%
Specialist‐involving	28.28%	17.55%	13.23%
Generalist‐specialist	7.77%	5.51%	5.32%
Others	12.16%	18.96%	16.5%
Negative edges			
Overall negative	38.43%	37.54%	43.36%
Generalist‐involving	8.73%	6.09%	17.15%
Specialist‐involving	16.43%	10.32%	9.45%
Generalist‐specialist	4.76%	4.37%	4.35%
Others	8.51%	10.72%	12.42%

*Note:* Edge percentages refer to either generalist or specialist involved relationships (i.e., generalist‐generalist, generalist‐others, or interrelated generalist‐specialist).

The networks at either salinity extreme showed increased modularity and clustering coefficients (Table 2; Figure S9A,C), especially among specialists, suggesting the formation of tightly knit sub‐communities adapted to hypersaline conditions. This specialisation may enhance community efficiency and stability under extreme conditions.

At the node‐level, generalists dominated in terms of centrality measures (a measure of how nodes act as connectors within the community) at lower salinity levels, indicating they act as connectors within the community (Figure S9A). However, this distinction diminished as salinity increased, and specialists became more prominent in the network (Figure S9B,C). The convergence in clustering metrics between generalists and specialists at intermediate salinity suggests increased interactions between these groups, potentially reflecting a blending of roles or adaptation strategies (Figure S9B).

## 4 Discussion

This study revealed that salinity is a critical driver of microbial community composition, diversity and deterministic community assembly in sediments of the Coorong. The pronounced salinity gradient influences microbial communities by selecting for taxa adapted to specific salinity conditions, leading to the selection of more homogeneous communities at high salinity levels. Despite their increased homogeneity, increased competition and complex interactions were observed under hypersaline conditions.

### 4.1 Salinity‐Driven Deterministic Assembly of Microbial Communities

Salinity is widely recognised as the most important factor shaping microbial communities on global and regional scales (Caporaso et al. 2011; Logares et al. 2013; Lozupone and Knight 2007). As salinity increases, the thermal capacity of water decreases, meaning less heat is required to raise its temperature (Javor 1989). Additionally, pH decreases due to reduced dissociation of bicarbonate at high salinity levels (Krumgalz 1980). While water thermal stress (temperature) and pH significantly affect microbial community structure in the Coorong, they are likely secondary effects of salinity, introducing multiple environmental covariates. Consistent with these results, recent work in the Coorong showed flood‐driven freshening coincided with rapid shifts in both microbial communities and their metabolite profiles, suggesting salinity restructures communities and their biogeochemical function (Keneally, Chilton, et al. 2025).

Deterministic processes, particularly homogeneous selection, dominated community assembly in Coorong sediments, with their influence increasing along the salinity gradient. This aligns with previous studies showing that salinity acts as a strong environmental filter, promoting deterministic assembly in various environments (Yu et al. 2022; Zhang et al. 2021), as ionic strength rises and dispersal limitation weakens, especially in contiguous systems, supporting the patterns observed in the present study (Menéndez‐Serra et al. 2023; Song et al. 2022).

Recent studies in lake and river systems have found that temperature and flow fluctuations throughout the year rearranged the proportion of selection and drift pressures (Fang et al. 2023; Wenbao et al. 2024), which may be relevant to the Coorong's seasonal freshening dynamics. Other considerations in addition to salinity include how anthropogenic stressors (e.g., nutrients, metals, hydrocarbons) impose selective filters in coastal sediments that favour taxa with specific stress‐tolerance traits, further increasing the deterministic signal and reshaping interaction networks (Ramljak et al. 2025).

The steeper decline in specialist community similarity with geographic and salinity differences (DDR slopes) indicates that specialists were more sensitive to environmental differences, and salinity exerted a stronger filtering effect on them. Steeper spatial scaling has been associated with deterministic assembly mechanisms (Du et al. 2021), supporting the hypothesis that selection intensifies with increasing salinity along the spatial salinity gradient in the Coorong. As the salinity thresholds for certain species are exceeded, their ecological functions may be taken up by other groups, resulting in shifts in community functions in response to environmental stress (Martiny et al. 2015).

Stochastic community assembly declined with rising salinity in this study, contradicting Song et al. (2022), which associated salinity with greater stochasticity in saltern ponds. The apparent conflict may reflect contrasting spatial connectivity: solar saltern ponds are discrete, separated and highly dispersal‐limited (Song et al. 2022), whereas the Coorong is a continuous water body with a steep, continuous salinity gradient. As detailed by Chilton (2024), frequent wind‐driven resuspension and sediment mixing occur in the Coorong, likely reducing dispersal limitation among microbes here, allowing for strengthened environmental selection and thus lower stochasticity.

Salinity was the strongest correlate of diversity and assembly signals, but it likely tracks a coupled physicochemical regime (e.g., oxygen/redox, nutrients/organic matter, residence time) rather than acting alone. We therefore interpret patterns as salinity‐associated selection, with causality requiring targeted tests, including direct experimental manipulation or designs that decouple salinity from co‐varying drivers.

### 4.2 Specialist and Generalist Taxa Responses

Specialist taxa demonstrated more pronounced responses to salinity variations compared to generalists. The significant reduction in diversity among specialists at high salinity levels indicates that only a limited number of taxa can thrive under such extreme conditions. Key specialists identified in hypersaline conditions included members of Halanaerobiaeota such as *Halanaerobium* spp., which are obligate fermentative anaerobes known to accumulate KCl for osmoadaptation (Oren 2008). These organisms have roles in bioremediation of contaminants under high‐salt conditions (Roush et al. 2014), and are prevalent in diverse saline environments, including shale oil wells (Lipus et al. 2017). Other important specialists included salinity‐adapted *Balneolaceae* and Archaeal halophiles from the phylum Halobacterota (Swan et al. 2010). Some specialists were abundant at both salinity extremes, including the underexplored bacterial phylum Gemmatimonadota group PAUC34f, typically found in coastal sediments and harbouring genetic potential for biosynthesis of secondary metabolites including antifungals, antibiotics and immunosuppressants with pharmaceutical potential (Gong et al. 2024).

Several taxa of biogeochemical relevance were identified as key hypersaline specialists. Examples include *Desulfopila* and *Desulfotignum*: important sulfate‐reducers in hypersaline habitat (Li et al. 2016; Selak et al. 2025; Suzuki et al. 2007), and Methanomassiliicoccales, recently identified as a critical methanogen in the Coorong (Keneally, Chilton, et al. 2025; Keneally, Southgate, et al. 2024). Contrastingly, most members of the *Sulfitobacter* genus, here identified as important estuarine specialists, are implicated in sulfur oxidation processes and were highly abundant in low salinities (Sorokin 1995; Xu et al. 2024). These results suggest that salinity‐driven changes in sulfur and carbon cycling favour sulfur oxidation at lower salinity and sulfate reduction and methanogenesis in hypersaline habitats. In the same system, metabolomic analyses found hypersaline sediments enriched in osmoprotectants (e.g., glycine betaine/choline) and associated taxa, with freshening linked to broader shifts consistent with changes in sulfur and nitrogen cycling (Keneally, Chilton, et al. 2025). Despite the importance of specialists, generalists like *Oleiphilus* have been identified as significant in hydrocarbon degradation related to the Deepwater Horizon oil spill (Hazen et al. 2010). During environmental fluctuations in dynamic salinity zones, the increased importance of generalists may contribute to ecosystem resilience (Walker et al. 2022; Xu et al. 2022). Generalists can maintain essential functions when specialist diversity declines, buffering ecosystems against disturbance (Dehling et al. 2021; Richmond et al. 2005).

### 4.3 Microbial Community Network Adaptation to Salinity Stress

Network analyses suggest that microbial communities become more interconnected, specialised and competitive at higher salinity levels. The rise in negative edges (Section 3.6) indicates a putative shift from collaboration to competitive exclusion, consistent with recent metabolomic evidence of intensified osmolyte production that limits freely shared substrates (Keneally, Chilton, et al. 2025). The observed increases in competition and connectivity imply that Coorong sediment microbial communities reorganise their interactions to optimise survival under salinity stress. This aligns with previous studies, where microbial competition and network complexity typically increase with salinity (Escalas et al. 2021; Ji et al. 2019; Ya et al. 2022). These results should be interpreted with care, as network inference approaches may be sensitive to strain‐level genomic variation and spurious inter‐taxa correlations, especially in habitats with high heterogeneity (Berry and Widder 2014). This study attempted to reduce these effects by following best practices outlined by Berry and Widder (2014).

As the salinity tolerance of taxa changes, communities adjust their interactions, potentially affecting ecosystem processes such as nutrient cycling and energy flow (Escalas et al. 2021). The increased modularity and clustering at salinity extremes may represent the formation of alternate stable states, where microbial communities adopt different configurations optimised for specific environmental conditions (Beisner et al. 2003; Hu et al. 2022). This agrees with previous research conducted in inland salt lakes of Xinjiang, China, where microbial communities conform to alternate stable states at salinity extremes, with destabilisation under intermediary freshening and salinisation (Hu et al. 2022).

Despite salinity‐driven decreases in α‐diversity, the importance of specialists in networks at salinity extremes may suggest competitive advantages in stable environments, where specialists efficiently utilise available resources. In contrast, generalists possess broader ecological niches and greater tolerance to environmental fluctuations, aligning with prior research (Matias et al. 2013; Villalba et al. 2022; Zhou, Shen, et al. 2024). In dynamic salinity zones, regular disturbances prevent specialisation, promoting the persistence of generalists and increasing species diversity. This aligns with macro‐community ecology research (Devictor and Robert 2009; Leroy et al. 2014), and underlines the importance of niche specialisation‐based assessments in community ecology. Such assessments improve detection of habitat disturbance effects on communities (Devictor and Robert 2009), but this approach is relatively underutilised in microbial ecology.

### 4.4 Ecological Implications and Resilience

The differing responses of specialists and generalists to salinity stress have important ecological implications. The dominance of heterogeneous deterministic assembly processes and increased importance of specialists in hypersaline conditions suggest that these communities may be more susceptible to environmental disturbance. Determinism has previously been observed to vary with seasonality in eutrophic rivers and estuaries (Zhou, Wu, et al. 2024; Zhou et al. 2021).

Understanding microbial dynamics across salinity gradients can enhance management and conservation of hypersaline coastal lagoons like the Coorong. Because specialists underpin key processes such as sulfate reduction and methane production, their vulnerability implies that sudden freshening (e.g., managed freshwater releases or flooding) could transiently disrupt the production of methane and toxic sulfides before more resilient generalists re‐establish ecosystem functions (Keneally, Chilton, et al. 2025). Managers could therefore pair salinity‐mitigation flows with monitoring of microbial community succession, functional metabolites (e.g., osmoprotectant compounds; Keneally, Chilton, et al. 2025), and greenhouse‐gas emissions to detect ecosystem benefits. Furthermore, understanding and predicting microbial responses to salinity change is essential for forecasting the impacts of climate change and human activities on these globally important ecosystems (Keneally, Gaget, et al. 2025).

## 5 Conclusion

Salinisation in the Coorong lagoon drives increased selection of specialised microbial communities, resulting in decreased diversity but heightened competition and complex interactions among the remaining species under hypersaline conditions. In dynamic salinity zones, regular disturbances prevent specialisation, promoting diversity and resilience of generalist taxa. Furthermore, this study identifies putatively important functional taxa specialised for hypersaline conditions, particularly sulfate‐reducing Bacteria (e.g., *Desulfotignum*, *Desulfopila*) and methanogenic Archaea (e.g., *Methanomassiliicoccales*), which may be vulnerable to salinity reduction. Consequently, flooding or targeted freshening of degraded hypersaline areas could transiently alter methane and toxic sulfide dynamics, while creating conditions that favour sulfur‐oxidising bacteria (e.g., *Sulfitobacter*) that can help detoxify sediments, and more resilient generalists that buffer core functions across intermediate salinities as communities re‐establish. While this work offers detailed insight into community structure, and the hypothesised functional roles are supported by metabolomic and multi‐omic studies from the same system, these putative functions should be validated with direct functional gene or pathway measurements to strengthen causal links between community composition and biogeochemical processes.

## Author Contributions

Conceptualization: C.K., V.G., A.E.K., and J.D.B. Methodology, formal analysis and project administration: C.K. Investigation: C.K., D.C., T.N.D. Visualization: C.K., A.E.K., T.N.D., and J.H. Writing – original draft: C.K. and A.E.K. Writing – review and editing: V.G., D.C., T.N.D., J.H., A.E.K., S.P.K., and J.D.B. Supervision: V.G., S.P.K., and J.D.B. Funding acquisition: J.D.B.

## Funding

This work was supported by Medical Research Future Fund, 2024316.

## Conflicts of Interest

The authors declare no conflicts of interest.

## Supporting information


**Data S1:** emi470301‐sup‐0001‐Supinfo.pdf.

## Data Availability

Raw 16S rRNA (V3‐V4) amplicon sequences are available in the NCBI Sequence Read Archive under accessions: PRJNA1284436 and PRJNA1198852. Sample metadata is available in the Supporting Information.
