# Supplementary material for: Extreme Salinity Change Governs Microbial Community Assembly and Interactions
Source: Environ Microbiol Rep. 2026 Feb 15;18(1):e70301. doi: 10.1111/1758-2229.70301 (PMC12907032; doi:10.1111/1758-2229.70301)
Supplement: Supplementary file 1 — Data S1: emi470301‐sup‐0001‐Supinfo.pdf. [file EMI4-18-e70301-s001.pdf]

Supporting information for: Extreme Salinity Change Governs Microbial Community Assembly and Interactions

Christopher Keneally (ORCID: 0000-0001-7261-6259) [*1],

Virginie Gaget (ORCID: 0000-0001-5331-8600) [2, 3],

Daniel Chilton (ORCID: 0000-0001-8901-0536) [4],

Tyler N. Dornan (ORCID: 0000-0002-4998-7577) [1],

James Hensel (ORCID: 0009-0005-0348-4308) [1],

Ashleigh E. Keneally (ORCID: 0000-0003-2549-8741) [1],

Stephen P. Kidd (ORCID: 0000-0002-2118-1651) [1, 5, 6],

Justin D. Brookes (ORCID: 0000-0001-8408-9142) [1]

[1]School of Biological Sciences, Faculty of Science, Engineering and Technology, University of Adelaide, South Australia, Australia

[2]Discipline of Surgery, The University of Adelaide, The Queen Elizabeth Hospital, Adelaide, South Australia

[3]Basil Hetzel Institute for Translational Health Research, Central Adelaide Local Health Network, Woodville, South Australia

[4]College of Science and Engineering, Flinders University, South Australia, Australia

[5]Research Centre for Infectious Disease (RCID), The University of Adelaide, Adelaide, South Australia, Australia

[6]Australian Centre for Antimicrobial Resistance Ecology (ACARE), The University of Adelaide, Adelaide, South Australia, Australia

[*]Corresponding author

Table S1. Bottom water variation in environmental parameters between sites and seasons.

	Sample Count				Seasonal Salinity (g L^{-1})				Seasonal Temperature (°C)				Seasonal DO (mg L^{-1})				Seasonal pH			
**Site**	**Sum**	**Aut**	**Win**	**Spr**	**Sum**	**Aut**	**Win**	**Spr**	**Sum**	**Aut**	**Win**	**Spr**	**Sum**	**Aut**	**Win**	**Spr**	**Sum**	**Aut**	**Win**	**Spr**
**Long Point (LP)**[‡ ¶]	5	3	3	6	13.2 ± 7.72	15.4 ± 1.39	14.3 ± 0.22	25.3 ± 3.59	20.9 ± 5.46	19.0 ± 0	15.8 ± 0.12	16.57 ± 0.84	6.66 ± 1.43	9.0 ± 1.0	7.94 ± 0.04	9.59 ± 2.48	8.12 ± 0.27	7.83 ± 0.06	8.1 ± 0.1	8.42 ± 0.33
**Noonamena (NM)**[*‡]	3	-	-	-	31.49 ± 0.2				17.09 ± 0.68				11.81 ± 0.12				8.23 ± 0.01			
**Parnka Point (PA)**[*‡§]	5	-	-	-	81.83 ± 1.56				17.5 ± 1.37				5.43 ± 1.15				8.09 ± 0.06			
**Villa Dei Yumpa (VDY)** [‡§¶]	7	3	3	3	95.21 ± 19.5	118.55 ± 0.51	74.02 ± 0.08	58.65 ± 0.18	21.23 ± 5.25	19.3 ± 0	16.23 ± 0	17.07 ± 0.01	5.65 ± 0.92	4.92 ± 0.23	4.76 ± 0.15	7.4 ± 0.1	8.13 ± 0.13	7.8 ± 0	8.3 ± 0	8.53 ± 0.03
**Woods Well (WW) ¶**	3	3	3	3	91.82 ± 0.06	127.0 ± 0.85	73.6 ± 0.06	64.2 ± 0.2	24.73 ± 0.2	19.4 ± 0	16.21 ± 0	15.08 ± 0.36	5.18 ± 0.16	4.0 ± 0.4	4.77 ± 0.09	6.56 ± 0.09	8.17 ± 0.02	7.9 ± 0	8.34 ± 0.01	8.56 ± 0.01
**Jack Point (JP)**[†]	3	-	-	3	86.77 ± 0.68			77.12 ± 5.64	17.71 ± 0.65			17.13 ± 1.69	6.25 ± 0.01			6.19 ± 0.79	7.96 ± 0.02			8.18 ± 0.12
**Policeman Point (PP)**[§¶]	5	3	3	3	106.74 ± 13.97	130.16 ± 0.6	79.15 ± 1.3	70.79 ± 0.07	21.48 ± 2.99	19.1 ± 0	15.9 ± 0.03	18.11 ± 0.17	4.99 ± 0.4	3.95 ± 0.17	5.11 ± 0.17	7.23 ± 0.12	7.97 ± 0.2	7.9 ± 0	8.28 ± 0.02	8.61 ± 0.03
**Snipe Point (SPT)** [¶]	3	3	3	3	84.96 ± 0.22	129.87 ± 0.43	82.15 ± 0.18	69.18 ± 0.34	25.52 ± 0.25	18.8 ± 0	14.27 ± 0.01	17.33 ± 0.22	5.47 ± 0.42	4.2 ± 0.26	4.64 ± 0.32	6.48 ± 0.03	8.23 ± 0.01	8.1 ± 0	8.32 ± 0.01	8.23 ± 0.01
**Salt Creek (SC)** [‡§¶]	7	3	3	3	103.88 ± 9.0	141.74 ± 0.43	85.69 ± 0.1	75.05 ± 0.02	20.21 ± 1.98	19.0 ± 0	14.27 ± 2.88	15.67 ± 0.08	5.19 ± 0.57	3.5 ± 0.3	6.35 ± 0	5.94 ± 0.02	7.07 ± 1.11	8.3 ± 0	8.29 ± 0.03	8.48 ± 0.05

Values are expressed as mean ± standard deviation. Values include samples from 2019, 2021, and 2022. [*]Site sampled only in summer, [†]site sampled only in summer and spring, [‡]includes 2019 samples, [§]includes 2021 samples, [¶]includes 2022 samples. Sum: Summer, Aut: Autumn, Win: Winter, Spr: Spring.

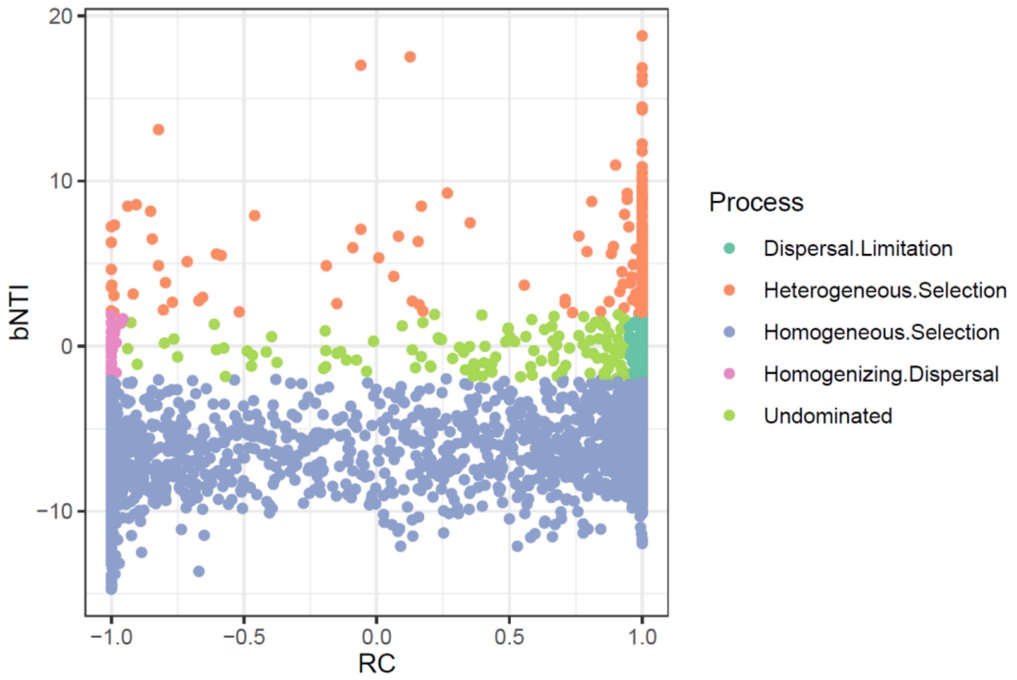

Figure S1. Scatterplot conceptualising null model-based community assembly determination. The beta-nearest taxon index (βNTI) of pairwise community comparisons identifies deterministic community assembly, where significant deviation from null values occurs (heterogeneous selection: |βNTI| > 2, or homogeneous selection: |βNTI| < -2). The RC_{bray} index further categorises stochastic assemblies falling between |βNTI| -2 and 2. These include dispersal limitation (RC_{bray} > 0.95), homogenising dispersal (RC_{bray} < -0.95), and undominated processes not significantly deviating from the null (RC_{bray} between -0.95 and 0.95).

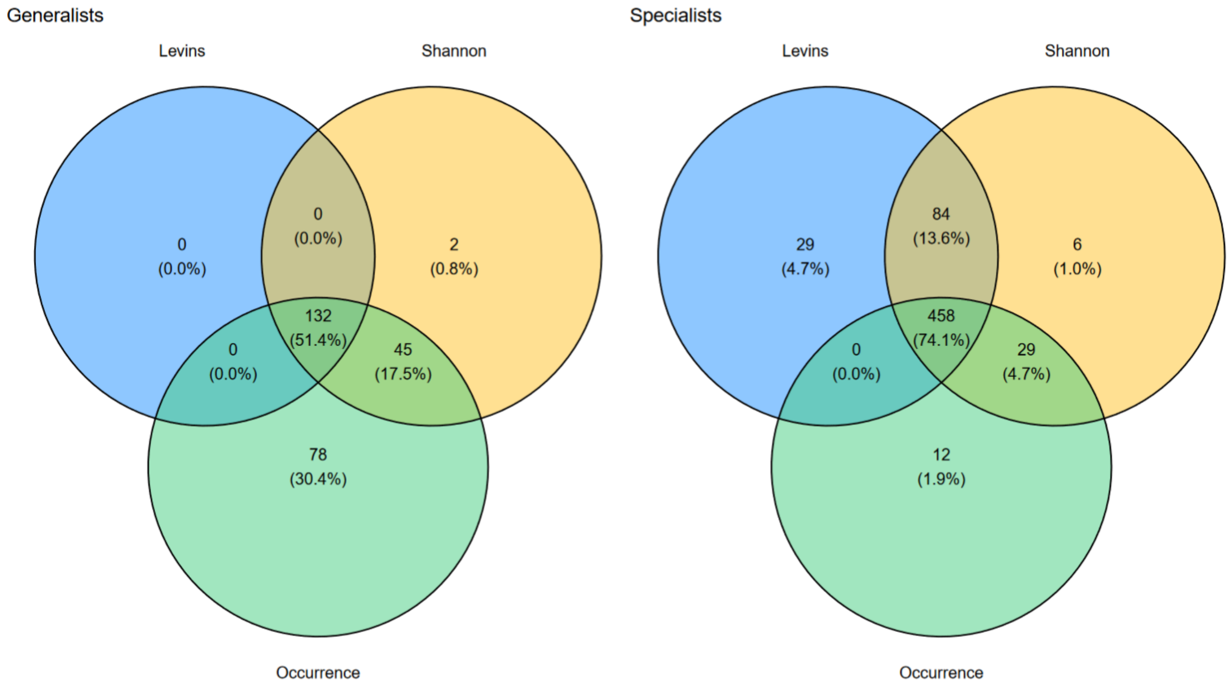

Figure S2. Venn diagram of taxa classified as 'Specialists' or 'Generalists.' Taxa were classified based on a combination of 1) Levins' niche index, 2) Shannon diversity, and 3) occurrence. Taxa were finally classified if all 3 methods agreed. Integers represent number of taxa classified with percentages of total taxa below.

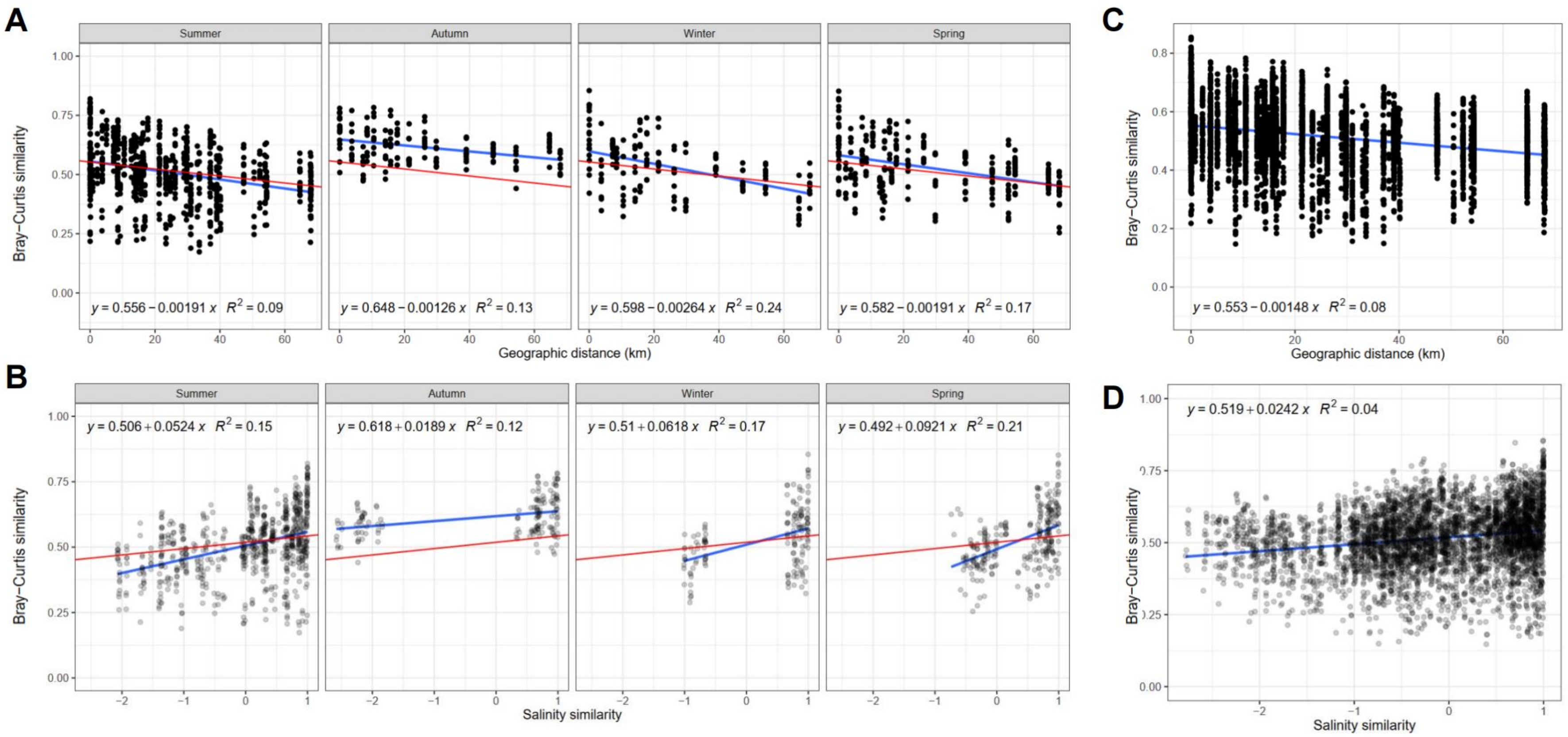

Figure S3. Generalist community similarity, based between-sample Bray-Curtis distances, regressed against spatial distribution and salinity similarity. A) Spatial distribution and **B) salinity similarity** faceted by season. **C) Spatial distribution** and **C) salinity similarity with all seasons combined.** Blue regression lines show linear fit for subset, while red lines indicate the full regressions shown in C) and D). All derived linear models pass hypothesis testing with *p*-values not exceeding 5.67×10^{-6}.

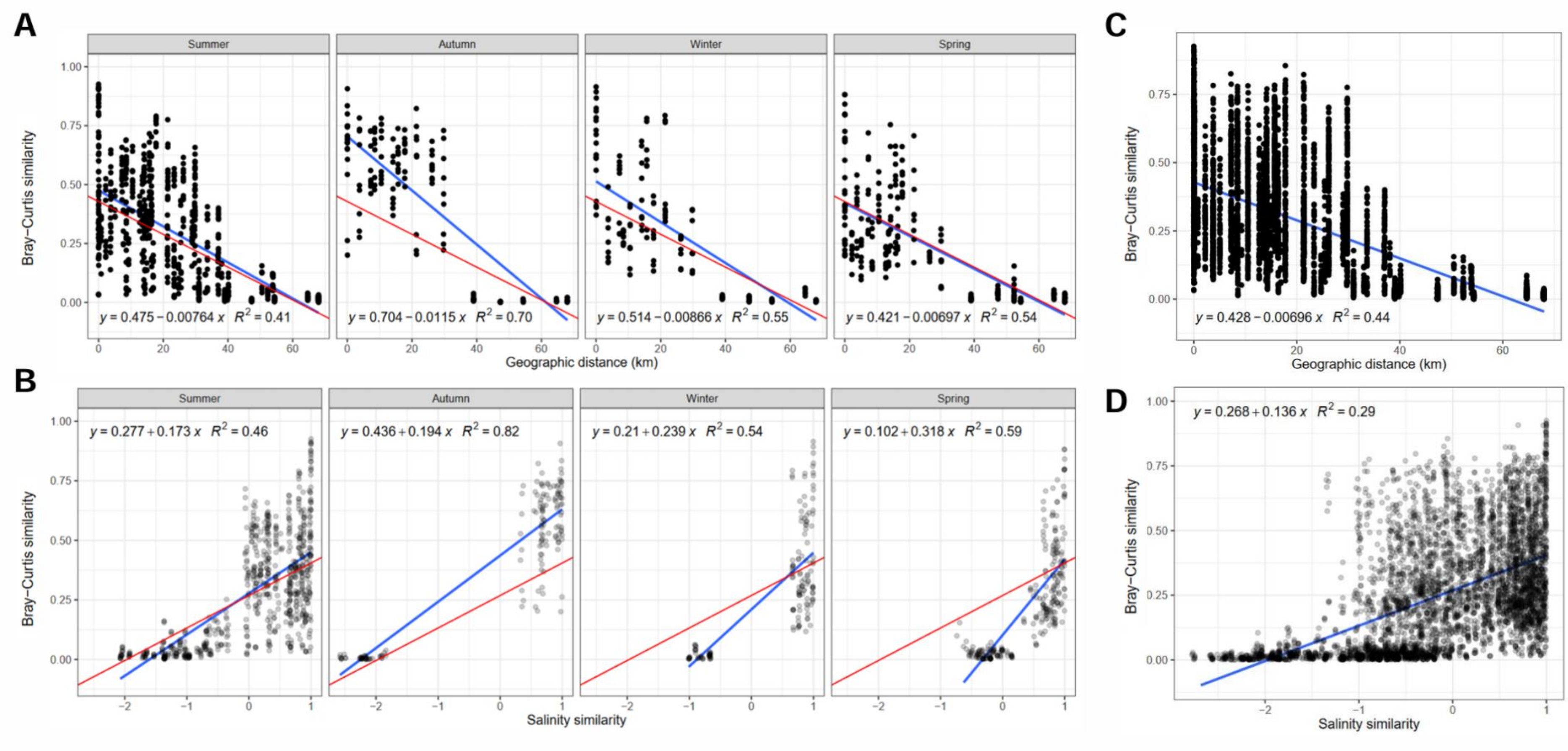

Figure S4. Specialist community similarity, based between-sample Bray-Curtis distances, regressed against spatial distribution and salinity similarity. A) Spatial distribution and **B) salinity similarity** faceted by season. **C) Spatial distribution** and **C) salinity similarity with all seasons combined.** Blue regression lines show linear fit for subset, while red lines indicate the full regressions shown in C) and D). All derived linear models pass hypothesis testing with *p*-values not exceeding 5.67×10^{-6}.

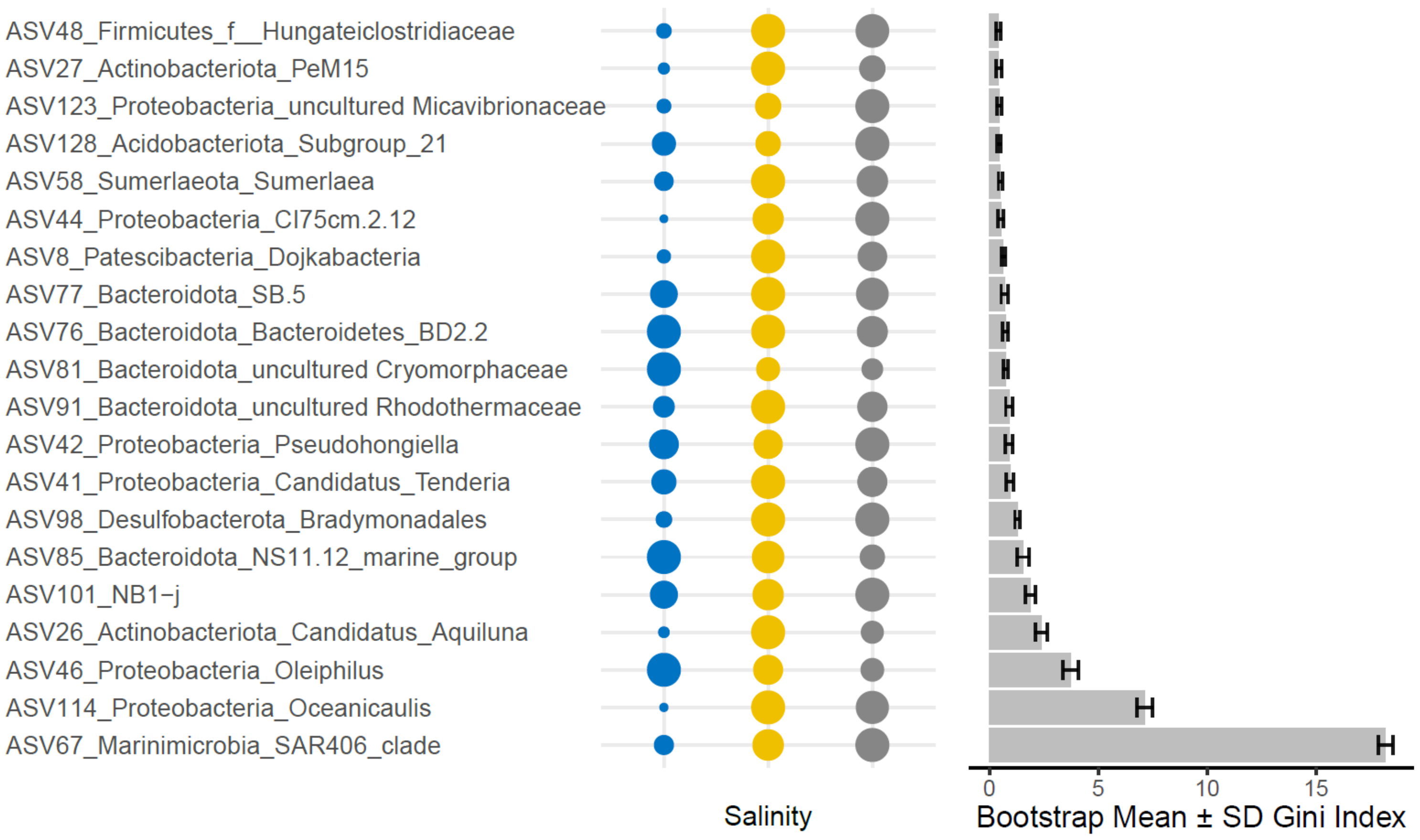

Figure S5. Top 20 Generalist taxa discriminating between salinity categories. For each taxon, taxonomy is displayed at both Phylum level and the deepest classified level. Dot plots indicate the relative abundance by salinity category (dot size is proportional to relative abundance per sample), distributed along the x axis and represented by colour, as follows: blue: 0-40 g L^{-1}, yellow: 40-100 g L^{-1}, grey: 100-150 g L^{-1}. The horizontal bar graph shows the mean importance of each taxon as predicted by bootstrapped random forest modelling.

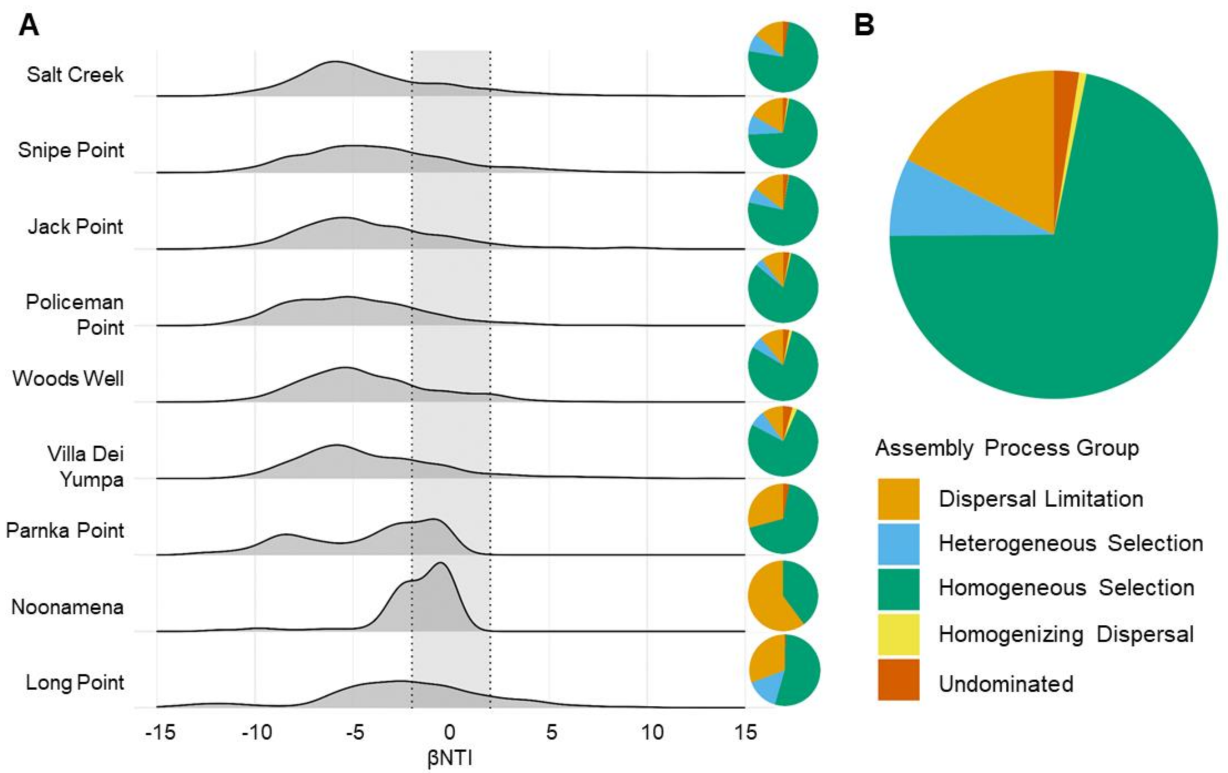

Figure S6. Community assembly processes structuring sediment microbial communities. A) Spatial variation in βNTI between sampling sites along the salinity gradient. B) Overall distribution of community assembly processes. Ridge plots display the distribution of βNTI across each sampling site, ordered from the Southernmost site (Salt Creek) to the Northernmost (Long Point). Shaded areas flanked by dotted lines indicate significant deviation of βNTI from null, while pie charts represent the relative contribution of stochastic and deterministic assembly processes.

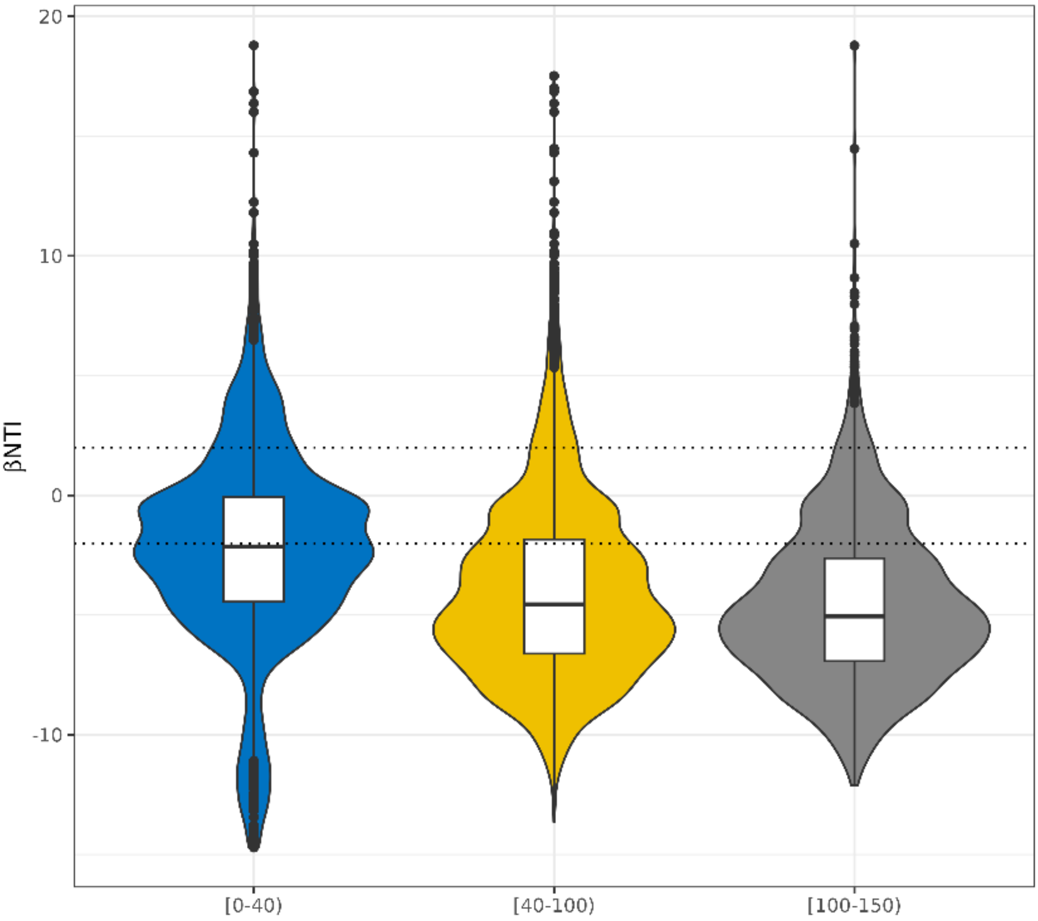

Figure S7. Violin plot of βNTI distribution by salinity bin. Dotted lines represent the point of significant difference from null, where βNTI < –2 indicates homogeneous selection in a community, while βNTI > 2 indicates hetereogenous selection of a community. Values not significantly different from null (within the dotted lines) indicate stochastic community assembly processes. Low salinity (0 – 40 g L^{-1}) is represented by blue, medium salinity (40 – 100 g L^{-1}) is represented by yellow, and high salinity (100 – 105 g L^{-1}) is represented by grey.

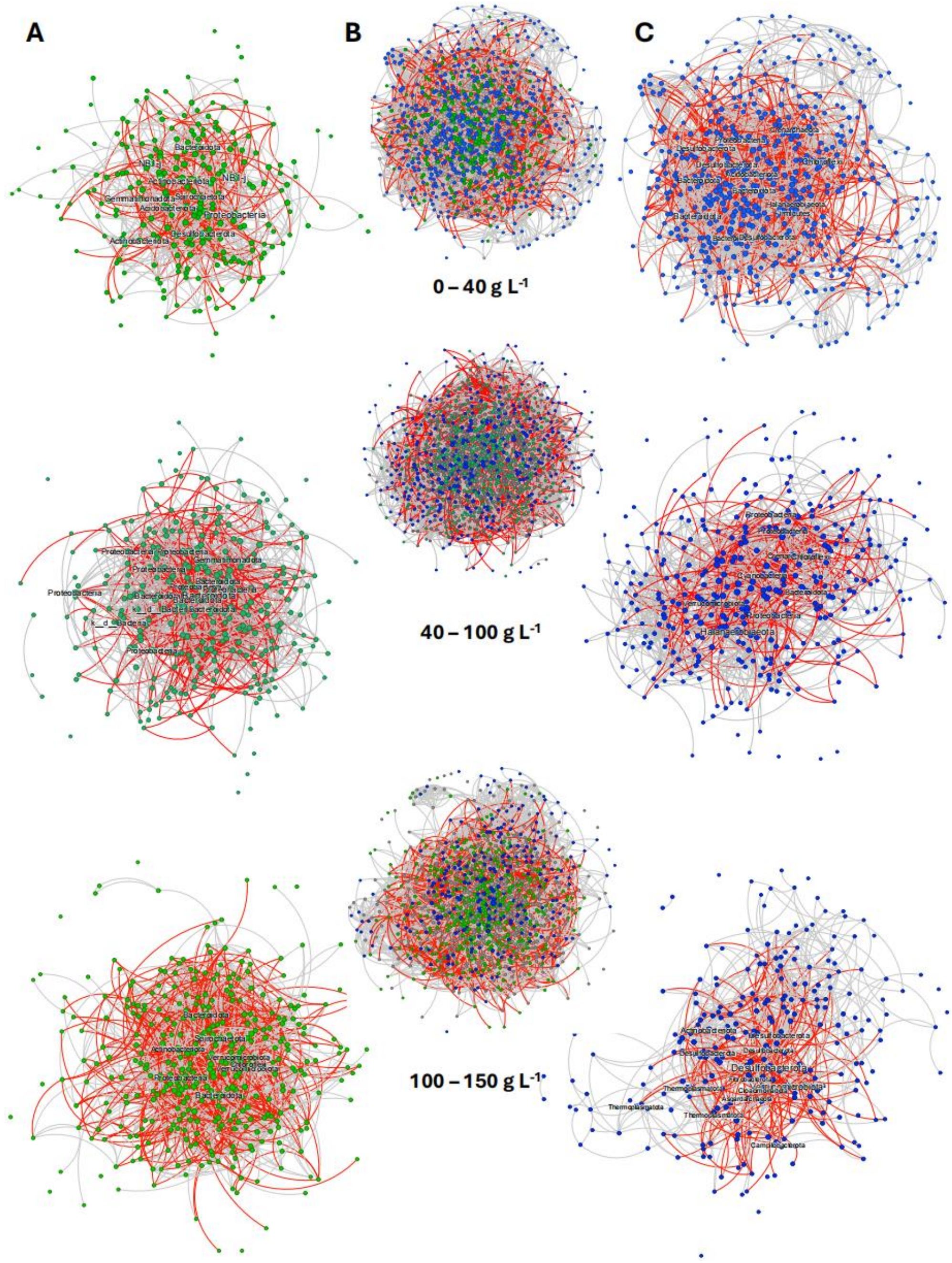

Figure S8. Microbial community subnetworks by salinity category. A) Generalist-filtered networks, **B) Whole networks**, and **C) Specialist-filtered networks.** Nodes are coloured by specialisation category. Green nodes: Generalists, Blue nodes: Specialists. Edges (connections between nodes) are coloured to indicate positive (grey) or negative (red) relationships between nodes. Taxa classified as neither generalist nor specialist are filtered from the visualisation for improved readability, but retained in network-level and node-level topology results. Highly connected nodes are labelled with Phylum names in **A)** and **C)**.

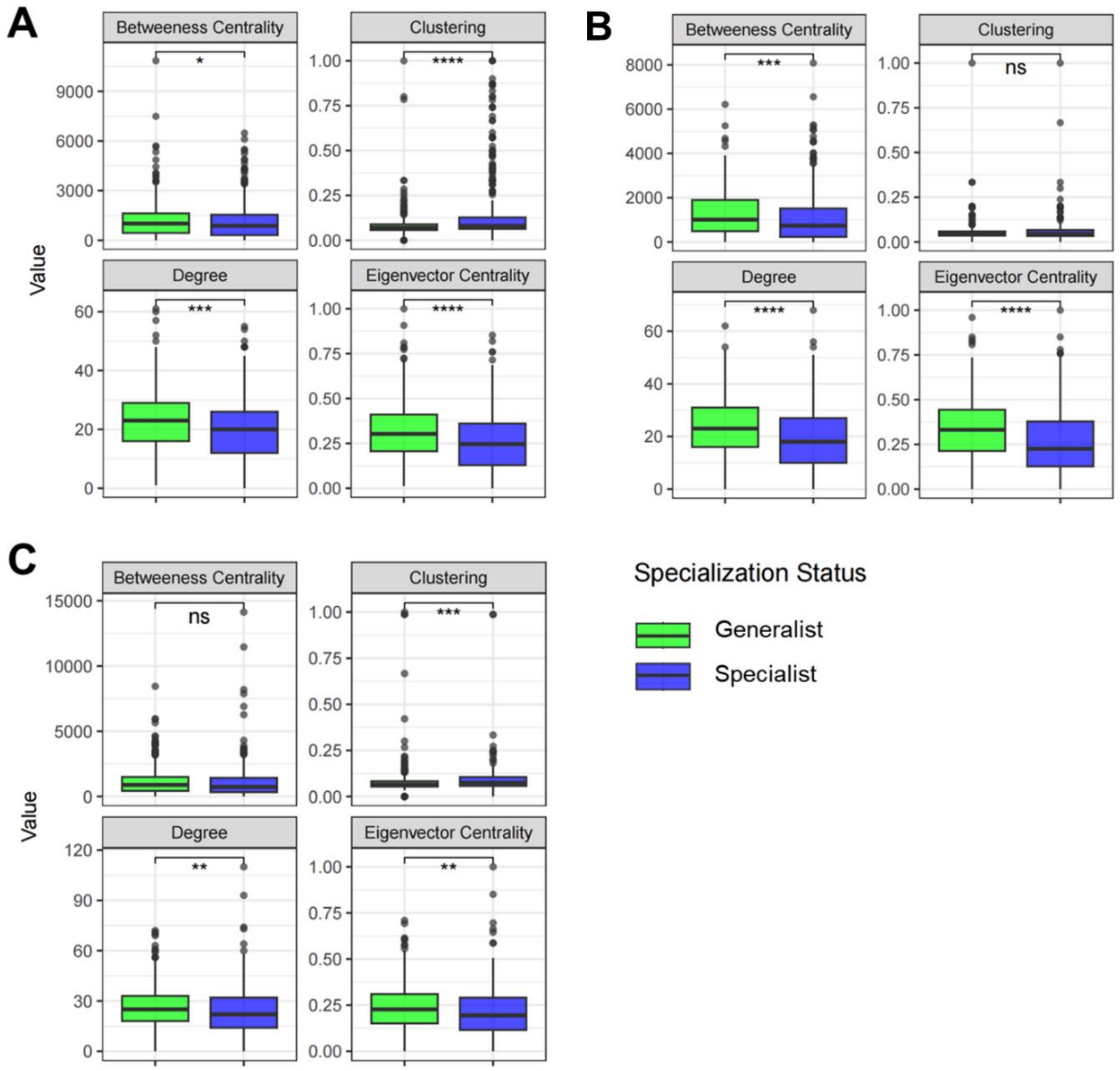

Figure S9. Boxplots visualising node-level statistics of topological microbial community network features. Facet grids are grouped by the salinity category of the corresponding subnetwork. **A) Low salinity (0 – 40 g L^{-1}) subnetwork, B) α-Hypersalinity (40-100 g L^{-1}), C) β-Hypersalinity (100 – 150 g L^{-1}).** Significant differences in groups were identified with Wilcoxon tests. *: $p < 0.05$, **: $p < 0.01$, ***: $p <0.001$, ****: $p < 0.0001$, ns: $p \geq 0.05$
